# Self-assessed knowledge of genomic medicine among non-genetics physicians – results from a nationwide Swedish survey

**DOI:** 10.1007/s12687-025-00818-y

**Published:** 2025-07-18

**Authors:** Joar Björk, Mikaela Friedman, Amy Nisselle, Maria Johansson Soller, Charlotta Ingvoldstad Malmgren

**Affiliations:** 1 Centre for Research Ethics & Bioethics, Uppsala University, Uppsala, Sweden; 2 Swedish National Centre for Priorities in Health, Linköping University, Linköping, Sweden; 3 Department of Research and Development, Region Kronoberg, Växjö, Sweden; 4 Department of Molecular Medicine and Surgery, Karolinska Institutet, Stockholm, Sweden; 5 Genomics in Society, Murdoch Children’s Research Institute, Melbourne, Australia; 6 Department of Paediatrics, The University of Melbourne, Melbourne, Australia

## Abstract

Genomic medicine is becoming essential to clinical everyday practice in many medical fields. At the same time ethical, economic and practical challenges impede the mainstreaming of genomic medicine. Previous studies indicate that physicians without specific qualifications in genetic medicine may lack sufficient knowledge and/or confidence to efficiently practise genomic medicine. This study used a previously validated survey to investigate Swedish non-genetics physicians’ (*n* = 133) self-assessed knowledge, education needs and preferred mode of working with genomic medicine. Self-assessed knowledge varied widely between knowledge areas, with about 70% having knowledge in basic concepts and disorders/diseases, to about 35% having knowledge about cascade testing or legal implications. A majority reported that having more knowledge within the field of genomic medicine would change their clinical practice. Only 3% of participants preferred to manage genomic medicine patients fully on their own, while 56% preferred to manage such patients in their own practise but with support from regional genetics services or competence in-house. In contrast, 29% preferred to refer patients directly to regional genetics services. A majority desired more or better access to information material aimed at physicians and/or patients. Answering patterns generally corroborate previous findings from other countries. In summary, Swedish non-genetic physicians report sub-optimal levels of knowledge and desire much support in genomic medicine matters. The results have important implications for the interaction between regional genetics services and non-genetics physicians, as well as for the continuous education necessary to further the mainstreaming of genomic medicine.

**Supplementary Information:**

The online version contains supplementary material available at 10.1007/s12687-025-00818-y.

## Background

To apply genomic medicine means to use an individual patient’s genotypic information in his or her clinical care (Manolio et al. 2013). It is quickly becoming an important part of clinical everyday practise in many medical fields (Stark et al. 2019a, b; Horgan et al. 2020). By using genomic medicine, clinicians are now able to, among other things, diagnose genetic conditions more quickly, make very detailed prognostications, and individualize dosage and target treatment. The advantages of this may extend far beyond individual patients, as insights from genomic medicine are applied to largescale screening and prevention programmes (DuBois et al. 2021).

The further development and implementation of genomic medicine is nonetheless associated with a diverse set of hurdles. There are numerous and complex ethical challenges in the practice of genomic medicine (see for instance Eichinger et al. 2021; Knoppers and Beauvais 2021; Smit et al. 2024), and the relatively high costs pose ethical and practical difficulties (Goranitis et al. 2021; Mwale and Farsides 2021; Girisha 2024). Furthermore, although studies indicate mainly positive attitudes to genomic medicine there are also patients and members of the general population who have hesitant or negative attitudes towards genomic medicine (Hassan et al. 2020; DuBois et al. 2021; Etchegary et al. 2021; Elphinstone et al. 2024; Harris et al. 2024; Pearce et al. 2024). The same goes for healthcare providers (Hauser et al. 2018; White et al. 2020). Last, there is much evidence of lack of knowledge on genomic medicine among all relevant stakeholders (Arora et al. 2016; White et al. 2020; Nisselle et al. 2021; Pysar et al. 2021; Fahim et al. 2024; Pearce et al. 2024).

The lack of knowledge of genomic medicine is, arguably, most alarming when it comes to healthcare providers, as these are the ones entrusted with providing healthcare– including genomic medicine. Indeed, genomic medicine is currently in a phase of “mainstreaming”, by which is meant that non-genetics physicians are increasingly expected to take on tasks within genomic medicine (White et al. 2020). For this mainstreaming to be successful, the level of “genomic literacy” among non-genetics physicians must be high, arguably far higher than empirical research indicates (Topol 2019; Nisselle et al. 2023). There are well described lacunae in medical education as well as in physicians’ continued education (Simpson et al. 2019; McClaren et al. 2020a, b; Johnson, Desalyn et al. 2022; French et al. 2023; Maher et al. 2023). Internationally established educational goals include: eliciting, documenting and acting on relevant family history; using genomic testing to guide patient management; using genomic information to make treatment decisions; as well as mastering pedagogical and emotional competencies necessary to interact with and support patients and relevant others (Tognetto et al. 2019; Whitley et al. 2020; Massingham et al. 2022; Mateo et al. 2022).

The present study sought to gain insights about the current state of mainstreaming of genomic medicine in Sweden, by surveying Swedish non-genetics physicians’ self-assessed knowledge and education needs regarding genomic medicine. The research questions were:

- What knowledge levels and knowledge gaps do non-genetics physicians identify in themselves?
- What information is available to non-genetics physicians and patients in the field of genomic medicine?
- What is the preferred role of non-genetics physicians in working with genomic medicine and what support do they require to be able to take on own patient responsibility?
- What forms of education and training are non-genetics physicians currently involved in, and what forms do they desire in the future?

## Methods

### Study design

This survey study was based on a previously validated survey (McClaren et al. 2020a)^1^. The survey was translated to Swedish and slightly modified to the context of Swedish healthcare delivery. Some questions were added, which sought to gain a deeper understanding of financing of genetic testing as well as participants’ view of patients’ knowledge of genomics. The Swedish version of the survey was pilot tested by five clinical geneticists and 11 non-genetics physicians. Surface validity was assessed through cognitive interviews with four non-genetics specialists. The finalized survey had up to 174 questions, depending on branching based on question responses. Categorical, rating and open-ended questions collected data on demographics, current and preferred genomic practice, and current and preferred genomics education and training (Supplementary Materials).

### Context and participants

The survey was deployed to Swedish non-genetics physicians. Swedish physicians who have a specialisation may belong to any (or a combination of) 63 different medical specialities, of which clinical genetics is one (Socialstyrelsen 2024). Clinical genetics can be a primary specialty or an add-on specialisation. The public healthcare system in Sweden is largely tax funded. Governance is decentralized with 21 county councils having responsibility for managing their own healthcare. Many private healthcare providers have contracts with county councils to operate within the tax funded system. Comprehensive clinical genetic service, i.e. service with both pre- and post-test counselling and genetic testing for rare diseases with more advanced technologies (whole exome or genome sequencing), is only available at the country’s seven University hospitals. Some basic testing is also performed by the pathology and clinical chemistry services at many smaller hospitals. E-consultations are becoming more common, and as a result even patients living far from the University hospitals today may benefit from genetic consultation. There is some variation regarding what genetic services are on offer in the different regions. For instance NIPT has not been available all over the country but is gradually becoming more common. Whole genome/exome sequencing is now routine across the country. Each care unit has a specific budget for testing with no specific adjustment for more expensive laboratory investigations, such as genetic testing. Patients do and can only pay a fee for the medical appointment, but all further investigation including genetic testing is paid by the healthcare system.

Recruitment was via invitations in the Genomic Medicine Sweden Newsletter, on the organisation’s webpage and social media channels, as well as in the Swedish Medical Association’s Journal. Invitations were also sent to the specialist physicians’ associations for further dissemination. Participants were encouraged to invite further participants (snowball sampling) to reach study subjects expected to have insight and opinions on the topic. Exclusion criteria were: not being a physician; not seeing patients as part of one’s clinical practice; or having clinical genetics as primary specialty. The survey was administered online using KI-survey, a survey software developed at Karolinska Institutet. The survey was open from Nov 8th 2021 to Oct 18th 2022.

### Data cleaning and analysis

Data were downloaded, cleaned and managed in Excel (Microsoft). Analysis included descriptive statistics and standard chi-2 test procedures using SPSS software (SPSS Inc). A p value of < 0.05 was considered significant. Due to the relatively scarce material, sub-group analysis was kept to a few factors including being a paediatrician/non-paediatrician (Eichinger et al. 2021). In sub-group analysis, “knowledge of basic concepts” was used as a proxy for other forms of knowledge. We used the definitions of engagement and confidence provided by the authors of the original survey (Nisselle et al. 2021). “Engaged” was defined as ordering a gene panel, exome or whole genome test within the last year. Being “overall confident” was defined as having an average score of “somewhat confident” or “very confident”, on a four-grade scale^2^ used to measure confidence across four core aspects of genomic medicine (knowledge about genomics, ability to elicit information about genetic conditions as part of a family or medical history, ability to explain genomic concepts to patients and ability to make decisions based on genomic information).

### Ethics

Ethical advice was sought from the Swedish Ethical Review Authority prior to commencing the study. In their answer (reg no. 2021–04311), the authority stated that they saw no major ethical concerns. All potential participants were informed that their participation was voluntary and that no sensitive information would be collected. Data was stored within the KI Survey instrument. All answers were anonymous at the point of using the survey software, but due to the relative low numbers of some specialists in some regions of Sweden, further measures were used to ensure that no participant could be identified. The survey commenced with the question of consenting to participate in the study and give permission for data to be used for research purposes.

## Results

### Demographics

Of 179 participants who commenced the survey, five opted out of including their data for publication and 41 were excluded as did not see patients in their practice, leaving 133 valid responses. This is thus the total number used in the Results section unless otherwise indicated due to stemming questions or missing data. Demographics are presented in Table 1. Physicians belonging to 37 specialities were represented, which is slightly more than half of all possible medical specialities in Sweden (Socialstyrelsen 2024). 41 participants had > 1 medical specialisation. The most common specialities were: Oncology (*n* = 17); General paediatrics (*n* = 16); Gynaecology and obstetrics (*n* = 15); Internal medicine (*n* = 14); Paediatric oncology (*n* = 10); Radiology (*n* = 10). Participants represented all geographical areas of Sweden, with a preponderance of participants working at university hospitals (83.5%).

**Table 1:** Demographics of the sample

Gender	
Female	73/133 (54.9%)
Male	57/133 (42.9%)
Does not want to answer/missing	3/133 (2.3%)
Age	
34	11/133 (8.3%)
35–44	35/133 (26.3%)
45–54	51/133 (38.3%)
55–64	29/133 (21.8%)
> 65	6/133 (4.5%)
Career stage	
Trainee and advanced trainee	20/133 (15.0%)
Fellow	17/133 (12.8%)
Senior consultant	96/133 (72.2%)
Years since university medical exam (*median*)	1–48 YEARS (*23*)

### Self-assessed knowledge

68.4% (91/133) reported feeling somewhat or very confident regarding their own competence in at least one or more of the four core aspects of genomic medicine (see Methods section). Table 2 shows proportions of respondents reporting knowledge of specific topics within genomic medicine, and which they wish to learn more about. The majority (75.9%) have learnt basic concepts and wish to learn more (77.4%). The most popular topic that respondents wish to learn more about was ‘Emerging applications in genomic medicine’ (86.5%). 54.9% (73/133) reported that having more knowledge within the field of genomic medicine overall would change their clinical practice.

**Table 2:** Top five and bottom five categories of self-assessed knowledge and desire to learn more (*n* = 133) (*The full table*,* titled* Table 2*,* can be found in Supplement*)

	Reports having knowledge of	Reports desire to learn more
Basic concepts	75.9%	77.4%
Disorders and diseases	69.9%	83.5%
Current applications in genomic medicine	63.9%	84.2%
Recognising patients who may benefit from genomic testing	60.2%	70.7%
Emerging applications in genomic medicine	58.6%	86.5%
*(Table is truncated)*		
Ethical implications	43.6%	67.7%
Interpreting genomic test results	43.6%	63.2%
Psychosocial implications	39.8%	63.2%
Legal implications	35.3%	66.9%
Cascade testing	34.6%	53.4%

### Self-assessed education needs

A large majority 73.7% (98/133) felt they had had too little education on genomic medicine during their university training. However, most (81.9%, 109/133) opined that genomic medicine is suitable for continuous education rather than university training. 24.1% (32/133) reported wishing to increase their knowledge in *all* 21 genomic medicine topics, and 6.8% reported not wishing to increase their knowledge in any area. For the desire to increase knowledge in specific areas, see Table 2 above.

A large majority, 73.7% (98/133), had acquired new knowledge within the field of genomic medicine during the last year. Of those who had done so, 86.7% (85/98) had acquired new knowledge in more than just one way. The most common ways of acquiring new knowledge were “asking a colleague” which 60.2% (80/133) of participants had done, followed by participating in multidisciplinary team meetings (43.6%; 58/133), and collegiate seminars at the workplace and reading journals (38.3%; 51/133). Conversely, attending university courses or other forms of formalized courses was reported by less than 15%.

When it comes to acquiring new knowledge in other ways than participants had already used, the three most desired forms were attending online webinars and courses (51.9%; 69/133), external courses (46.6%; 62/133) and external seminars and conferences (44.4%; 59/133). Attending university courses was less desired (28.6%; 38/133). Many desired to participate in several forms of continuous education they were not currently part of, with a majority (51.1%; 68/133) desiring to participate in five or more forms of continuous education.

### Contact with regional genetics services and desired forms of support

In terms of how physicians wanted to manage genomic medicine patients in their clinical practice, the most common response (55.6%) was to prefer managing patients themselves but with access to support when needed, divided between a preference for support from regional genetics services (39.1%; 52/133) and (16.5%; 22/133) who would prefer support from competence in-house. Indeed, 57.1% (76/133) indicated that they would find it valuable to have an in-house co-worker with specific genetic competence, for instance a genetic counsellor (answering options were not mutually exclusive). Almost a third of participants (28.6%; 38/133) would prefer not to manage genomic medicine patients themselves at all but to refer the patient to their regional genetics services. Free text answers indicated that some participants felt that their regional genetics services should take full responsibility for all tasks related to genomic medicine. The support that participants most frequently desired included determining whether a genomic test is suitable and interpreting test results (both 63.9%; 85/133). Fewer participants desired support in discussing results with patients/families or eliciting patient consent (34.6% and 12.8%, respectively). A small fraction (3%; 4/133) would instead prefer to manage patients themselves without in-house or out-house support.

### Availability of relevant information

In regard to guidelines, less than half of participants 33.8% (45/133) reported being aware of any guidelines on genomic medicine in their specialty area. Those that were aware of guidelines referred to guidelines from the local, regional and national levels, the most common being national care guidelines. In contrast, only two participants referred to international guidelines. The availability of patient information was reported to be low, with only 21.1% (28/120) of participants reporting that they use such information in conversation with patients. 57.9% (77/133) reported a desire for more/better patient information material. Among those desiring patient information material, the favoured form of material was written information with a length of 1–2 pages (77.9%; 60/77). At the same time, 17.3% (23/133) answered that they know of material that they do not use because they do not know whether it is accurate or they feel insufficiently knowledgeable to use it.

### Subgroup analyses

Engaged physicians were more likely to have knowledge about basic concepts, partake in continuous education and to prefer to manage patients themselves rather than referring them to regional genetics services (*X*^*2*^ = 11.54; *p* <.001; *X*^*2*^ = 27.19; *p* <.001 and *X*^*2*^ = 11.16; *p* <.001 respectively). Physicians who reported having knowledge about basic concepts were more likely to prefer to manage patients themselves and to partake in continuous education (*X*^*2*^ = 6.17; *p* =.013 and *X*^*2*^ = 9.62; *p* =.002 respectively). Paediatricians were more likely to have knowledge about basic concepts and to prefer to manage patients themselves compared to other medical specialists (*X*^*2*^ = 5.56; *p* =.018; *X*^*2*^ = 6.96; *p* =.008; and *X*^*2*^ = 10.31; *p* =.001 respectively). Participants who believed that having more knowledge in genomic medicine would change the way they practise their work were more likely to express desire for more knowledge than physicians who did not hold this belief, to desire to learn about five or more genomic medicine topics and to want to participate in five or more forms of continuous education (*X*^*2*^ = 8.05; *p* =.005, *X*^*2*^ = 17.16; *p* <.001 and *X*^*2*^ = 11.69; p = < 0.001, respectively).

There were no statistically significant differences in gender or time since university exam regarding possessing knowledge of basic concepts, wanting to manage patients independently, desiring more knowledge or partaking in continuous education.

## Discussion

The results from this study indicate that Swedish non-genetic physicians desire to learn more about genomic medicine and express a high degree of desire for support in genomic medicine matters. Encouragingly, given support, a majority would prefer to manage genomic medicine patients themselves rather than to refer them to regional genetics services. A majority are currently acquiring new knowledge in the field of genomic medicine in some form, and many want to learn more. We will now discuss these results in depth, and compare them to previous international studies, particularly those using the same survey as our study with Australian physicians (Nisselle et al. 2021; Nisselle et al. 2023) and English physicians (Bishop et al. 2023). In the following, these will be referred to as “the Australian study” and “the UK study”, respectively.

That non-genetics physicians report low levels of self-assessed knowledge in genomic medicine is well known from a range of contexts (Hamilton et al. 2017; Johnson, Liza-Marie et al. 2017; Hauser et al. 2018; Simpson et al. 2019; Rahawi et al. 2020; White et al. 2020). In our study, there was only one area of knowledge (“basic concepts”) that more than 70% of participants reported being knowledgeable about. Although being knowledgeable correlated with clinical activity, even some engaged physicians reported lacking knowledge which may illustrate the perceived difficulty of the topic.

As can be expected, a majority of participants reported wanting to learn more about genomic medicine, although proportions wanting to learn more were slightly lower than in the Australian study and others (Johnson, Desalyn et al. 2022; Lopez Santibanez Jacome et al. 2022). Other studies show that physicians expect future physicians should have much higher genomic medicine knowledge than they themselves have (French et al. 2023). Indeed, 55% in this survey stated that having more knowledge would impact their clinical practice, a proportion which is comparable to the UK study and higher than in the Australian study. That those who have this belief are more motivated to learn more is in line with previous research (Simpson et al. 2019).

Proportions of Swedish physicians currently acquiring new knowledge about genomic medicine were higher than in the UK and Australia, and like in these countries informal education was much more common than formalized training. Like their international peers, Swedish physicians desired a mix of clinically integrated education, with a low proportion desiring formalized training, for instance through university courses. This may relate to difficulties taking time out from clinical responsibilities, but it should also be noted that clinical integration of learning is in line with current education recommendations (Korf et al. 2014; Simpson et al. 2019; McClaren et al. 2020a, b; Maher et al. 2023; Nisselle et al. 2024). Participants favoured online training, as did their UK peers (see also Simpson et al. 2019). Again, this could relate to the desire to integrate learning into busy clinical everyday practice. However, considering that participants generally reported a sub-optimal level of knowledge, it is surprising that most expressed a preference for their current *modus operandi* of learning. Roughly, participants seem to want to learn more about the topics they are already knowledgeable about, by using the same education methods they are currently utilising. These findings merit close consideration, so that educational campaigns are well aligned with both knowledge needs and methodological preferences of non-genetics physicians. The fact that many indicate that they would need support in determining whether a genomic test is suitable and interpreting test results may currently function as a road block to genetic mainstreaming. If non-genetics specialists feel they do not know enough about what tests are suitable, or how to interpret results, they may refrain from ordering tests. This suggests that support and training should focus on these skills.

When comparing answering patterns with the suggested teaching goals in genomic medicine (see introduction), it is striking that participants in our sample report low knowledge *and* low desire to learn about “soft” aspects such as ethical issues, as well as much higher desire to receive support in technical matters than in “soft” matters such as communicating with patients and families. This pattern in consistent with findings in the Australian study. It could be so that ethical, pedagogical and communicative competencies, being more general, are areas where non-genetics physicians feel they can rely on their general competencies. It may be feared, however, that (some) physicians may be victims of unconscious incompetence. Indeed, genomic medicine involves some particularly difficult communicative challenges (Arora et al. 2016; McClaren et al. 2020a, b; Kruse et al. 2022; Dunlop et al. 2023) as well as thorny ethical issues (Eichinger et al. 2021; Kruse et al. 2022; Winkler and Knoppers 2022). If Swedish non-genetics physicians are to manage genomic medicine patients themselves but with “technical” support, as answers suggest they prefer, the communicative challenges will remain with them. In light of a suggested (relative) disinterest in “soft” issues among non-genetics physicians, it becomes even more pressing that there are genetic counsellors who can take on some of the “softer” tasks (Patch and Middleton 2018, 2019). A point that merits further investigation is that UK and Australian data suggest physicians in these countries are more interested in learning about ethical, legal and communication aspects than participants in the Swedish study. One reason for this difference might be that the UK and Australia have more and better integrated genetic counsellors, who help to highlight the importance of ethics and other soft issues. All in all, then, one may worry that Swedish non-genetics physicians’ desired content and forms of education may not adequately equip them for improved genomic medicine practice.

As already noted, nearly two thirds of participants preferred to manage genomic medicine patients themselves (with support if needed), rather than referring the patient to regional genetics services. This proportion is larger than in several other studies where more non-genetics physicians prefer to refer their patients than take care of genomic tasks themselves (Stark et al. 2019a, b; Jayasinghe et al. 2021; Nisselle et al. 2021; Lopez Santibanez Jacome et al. 2022). Qualitative studies suggest that non-genetics physicians may value the continued contact with their patients, which they might lose if they were to refer all genomic medicine patients (Pasquier et al. 2022). The low proportion of non-genetics physicians preferring to manage patients entirely by themselves (3%) is comparable to international figures (see above). Indeed, genomic medicine may be particularly suitable for teamwork compared with many other medical fields (Best et al. 2021). In line with this, nearly six out of ten participants desired to have a co-worker with special genomic medicine competence, and “asking a colleague” was the most common way of learning about genomic medicine in our study as well as in the UK study. We think this supports the hypothesis that non-genetics physicians desire easy and close contact with the person(s) who provide support in genomic medicine matters, as discussed in articles about in-house “genomic champions” and having a “buddy” at the regional genetics services (Carroll et al. 2016; McClaren et al. 2020a, b; Truong et al. 2021; Mackley et al. 2025). While there may be fear that (some forms of) access to support may hamper the drive to learn genomic medicine (Paneque et al. 2017), others have spoken of a “progression model” whereby non-genetics physicians may gradually become less dependent on support as they become more familiar with genomic medicine (Nisselle et al. 2023). The influential UK Topol review advocates both lifelong training and the importance of easy access to qualified support (Topol 2019).

As in the UK, a majority of participants stated there are no guidelines in their area. Together with low levels of self-reported knowledge among physicians and high level of expressed need for support, this indicates that creating guidelines should be a high priority. Answers suggest that guidelines should preferably be in Swedish, and furthermore that also more/better material intended for patient use is also needed. In a small country such as Sweden, national production of guidelines and patient material is challenging as there are limited number of professionals that can provide this kind of material, and the division of healthcare in self-governing regions make coordination difficult. However, national efforts such as knowledge-driven guidelines, the implementation of European reference networks for rare diseases, and a National Center for rare diseases are underway. Furthermore Genomic Medicine Sweden, a government supported initiative for implementation of genomic medicine in healthcare, produces patient material. Also, it should be noted that answering patterns suggest that merely providing guidelines is not enough, as a significant number of participants report not daring to use guidelines as they feel they cannot assess their quality. For instance, it is important that guidelines are easy to understand and entrustable. When it comes to patient information material, there have been calls to democratize genomic medicine knowledge by educating the population (Topol 2019). One rationale is that the population are rapidly becoming involved in genomic medicine by accessing direct-to-consumer (DTC) tests (Shendure et al. 2019). It has been argued that the companies selling DTC tests should do the educating (Whitley et al. 2020), but this is unlikely to take the burden of education entirely of physicians’ shoulders.

### Methodological reflections

Strengths of this study include a robust number of participants from varying medical specialities, as well as the use of a previously validated survey. The study design enables interesting international comparisons, especially as we have used the same terminology (“engaged” and “confident”) as in the original publications. As in any self-assessment of competencies, participants may over- and underestimate their genomic medicine knowledge and answering patterns may not correctly represent true knowledge. Due to the length and detail of the survey one may assume that potential participants with low interest and/or knowledge in the topic were deterred from participating. The survey was kept open for almost one year, which may be considered a rather long time given the high pace of changes within genomic medicine. Some nuance may have been lost in the conversion from a ten-point-scale in the original to a four-point-scale in the Swedish version of the survey (see footnote 1). It should also be noted that the high percentage of participants working at university hospitals does not reflect average Swedish healthcare.

## Conclusion

This survey study of Swedish non-genetics physicians indicates that knowledge levels in genomic medicine may be low. Indeed, this seems to be the perception of the non-genetics physicians themselves. Many but not all are willing to develop their knowledge in genomic medicine through education and training, preferably through peer-to-peer interactions and online courses and webinars. Most would prefer to manage genomic medicine themselves with support from regional genetics services or in-house experts, although a considerable fraction would prefer to refer patients to regional genetics services. Nearly six in ten wish to have a colleague with genomic medicine competence. Participants express a desire for Swedish-language information that they can access for their own benefit or to show to patients.

The survey study generated a rich material and further analyses can be found in a forthcoming article. Areas for future studies includes comparing self-assessed and actual knowledge in this context, assessing whether non-genetics physicians actually possess the communication skills arguably necessary for qualified genomic medicine tasks, and investigating what determines physicians’ preferences for getting support in-house *vis a vis* from regional genetics services. Furthermore, the advantages and disadvantages of various genomic medicine support structures from a systems perspective need to be better understood. A Swedish model for continuous education in genomic medicine should be drafted, using insights from this study. International studies using the same survey adapted for local contexts should be encouraged, as this makes international comparisons possible.

In sum, this study suggests Swedish non-genetics physicians have already taken important steps towards the mainstreaming of genomic medicine, but that continuous education, support from regional genetics services and improved guidelines are needed to continue this important endeavour.

## Electronic supplementary material

Below is the link to the electronic supplementary material.

Supplementary Material 1

Supplementary Material 2

## Data Availability

The Survey (in Swedish) can be found in Supplementary Data. Data from the survey can be made available through the authors upon special request.

## Footnotes

1 The original survey (in English) is available here: https://www.frontiersin.org/articles/10.3389/fgene.2020.00059/full#supplementary-material.

2 The Australian survey instead used a ten-grade scale. The scale steps in the Swedish survey were: ”very unconfident”– ”somewhat unconfident”– ”somewhat confident”– ”very confident”.
