# Supplementary material for: Self-assessed knowledge of genomic medicine among non-genetics physicians – results from a nationwide Swedish survey
Source: J Community Genet. 2025 Jul 18;16(6):669–77. doi: 10.1007/s12687-025-00818-y (PMC12569223; doi:10.1007/s12687-025-00818-y)
Supplement: Supplementary file 1 — Supplementary Material 1 [file 12687_2025_818_MOESM1_ESM.docx]

## Avsnitt 1: Demografi

Detta avsnitt ställer frågor om dig och din medicinska utbildning, anställning och arbetsområden.

1. **Vad är ditt kön?**

- Man
- Kvinna
- Icke- binär
- Vill inte svara

1. **Vilken ålder har du?**

- 24 eller under
- 25-34
- 35-44
- 45-54
- 55-64
- 65 eller över

1. **I vilken region är du verksam?**

- Region Stockholm
- Region Uppsala
- Region Sörmland
- Region Östergötland
- Region Jönköpings län
- Region Kronoberg
- Region Kalmar län
- Region Gotland
- Region Blekinge
- Region Skåne
- Region Halland
- Västra Götalandsregionen
- Region Värmland
- Region Örebro län
- Region Västmanland
- Region Dalarna
- Region Gävleborg
- Region Västernorrland
- Region Jämtland Härjedalen
- Region Västerbotten
- Region Norrbotten
- Ej verksam i Sverige

1. **Träffar du patienter i din yrkesutövning?**
   - Ja
   - Nej > REDCap-kodningsinstruktioner går till UNDERSÖKNINGSSTOPP "Tack för ditt intresse för vår enkät, men den här undersökningen är endast för läkare som träffar patienter i sin verksamhet."
2. **Vad är din nuvarande tjänstenivå?** *Du kan fylla i flera alternativ.*
   - AT/Underläkare
   - ST-läkare
   - Specialistläkare
   - Biträdande överläkare
   - Överläkare
3. **Vilket år erhöll du din läkarexamen från universitet?**
4. **Vilken är din huvudsakliga medicinska specialitet? Om du har dubbel utbildning eller har slutfört avancerad utbildning i en annan specialitet ange detta i kommentarfältet nedan.
   Obs! Denna studie gäller INTE för dig vars primära specialitet är klinisk genetik.**

Akutsjukvård

Äldrepsykiatri

Allergologi

Allmänmedicin

Anestesi och intensivvård

Arbets- och miljömedicin

Arbetsmedicin

Barn- och ungdomsallergologi

Barn- och ungdomshematologi och onkologi

Barn- och ungdomskardiologi

Barn- och ungdomskirurgi

Barn- och ungdomsmedicin

Barn- och ungdomsneurologi med habilitering

Barn- och ungdomspsykiatri

Beroendemedicin

Endokrinologi och diabetologi

Geriatrik

Gynekologisk onkologi

Handkirurgi

Hematologi

Hörsel- och balansrubbningar

Hud- och könssjukdomar

Infektionssjukdomar

Internmedicin

Kardiologi

Kärlkirurgi

Kirurgi

Klinisk farmakologi

Klinisk fysiologi

Klinisk immunologi och transfusionsmedicin

Klinisk kemi

Klinisk mikrobiologi

Klinisk neurofysiologi

Klinisk patologi

Lungsjukdomar

Medicinsk gastroenterologi och hepatologi

Neonatologi

Neurokirurgi

Neurologi

Neuroradiologi

Njurmedicin

Nuklearmedicin

Obstetrik och gynekologi

Ögonsjukdomar

Onkologi

Öron-, näs- och halssjukdomar

Ortopedi

Palliativ medicin

Plastikkirurgi

Psykiatri

Radiologi

Rättsmedicin

Rättspsykiatri

Rehabiliteringsmedicin

Reumatologi

Röst- och talrubbningar

Skolhälsovård (medicinska insatser i elevhälsan)

Smärtlindring

Socialmedicin

Thoraxkirurgi

Urologi

Vårdhygien

Om annat, specificera

1. **Vilken kategori av patienter träffar du?** *Markera alla som är relevanta.*

- Individer som står inför familjebildning
- Gravida
- Barn
- Vuxna
- Om annat, specificera

1. **Har du en ytterligare medicinsk specialitet?** (drop down)
2. **Vem är din huvudsakliga arbetsgivare? Välj ett alternativ.** *Om ditt alternativ inte är listat, markera andra och ange nedan.*

*Om du har två lika anställningar, vänligen överväg att markera din kliniska tjänst som din primära roll. Om du har två likadana kliniska roller hos två arbetsgivare,*

*vänligen välj en.*

- Universitetssjukhus
- Regionalt sjukhus
- Privat sjukhus
- Primärvård (offentlig)
- Primärvård (privat)
- Privatklinik/privat specialistklinik
- Forskningsinstitut/universitet
- Myndighet eller motsvarande
- Patientorganisation eller patientstöd
- Praktiserar för närvarande inte inom min specialitet
- Om annat, specificera

1. **Har du någon ytterligare arbetsgivare?** *Markera alla som är relevanta.*
2. **Har ditt huvudsakliga arbete de senaste 12 månaderna utförts i stad med Universitetssjukhus?** *Välj ett alternativ.*

- Ja
- Nej

## Avsnitt 2: Genomik i praktiken - nuvarande och framtida

**13)** **Har du varit involverad i några forskningsprojekt relaterade till genomik under de senaste fem åren?**

• Ja

• Nej

• Jag skulle vilja men har inte haft möjlighet

**14) Vilken typ av forskningsprojekt var det? *Markera alla som gäller.***

- Laborativ
- Klinisk forskning
- Samhällsvetenskap (ELSI: Etiska, juridiska och sociala aspekter, utbildning, politik, etc.)
- Bioinformatik
- Om annat, specificera

**15) Har du i din yrkesroll tagit kontakt med en klinisk genetisk avdelning under de senaste 12 månaderna?**

• Ja : avslöja fråga 16-17.

• Nej: avslöja fråga 18.

• Minns inte

**16) Hur ofta? *Välj bara en***

• Dagligen

• Varje vecka

• Månadsvis

• Kvartalsvis

• En eller två gånger

• Vet ej

**17) Varför kontaktade du en klinisk genetisk avdelning? *Markera alla som gäller***

- För att beställa ett genetiskt eller genomiskt test
- Information om ett misstänkt genetiskt tillstånd
- Konsultation kring om och i så fall vilken typ av genetiskt eller genomiskt test som ska beställas
- Konsultation kring hur man remitterar patienten till den klinisk genetiska avdelningen
- Hjälp med genetisk vägledning före testet
- Hjälp med genetisk vägledning efter testet
- Om annat, specificera

**18) Av vilken anledning har du inte kontaktat en klinisk genetisk avdelning? *Markera alla som gäller.***

- Genetik och genomik är inte relevant för min praktik
- Jag har ännu inte behövt råd från en klinisk genetisk avdelning i min praktik
- Jag kan hantera mina patienter utan råd från en klinisk genetisk avdelning
- Jag är inte säker på hur jag ska kontakta min klinisk genetiska avdelning
- Jag har inte tillgång till ett kliniskt genetiskt team eller avdelning
- Om annat, specificera

**19) Beställer du relevanta genetiska tester som du anser behövs för utredning av**

**patient oavsett kostnad i din verksamhet?**

- Ja
- Ja upp till en specifik kostnad
- Nej
- Vet ej

**20) Upplever du att finansiering/kostnaden för genetiska tester hindrar dig att beställa**

**vissa tester?**

- Ja
- Nej
- Vet ej

**21) Finns det kliniska riktlinjer för tester med genomsekvensering kopplat till din specialitet?**

Dessa kan vara lokala, nationella eller internationella.

• Ja: gå till fråga 22.

•Nej

•Vet ej

**22) Vilken verksamhet tar fram dessa riktlinjer (t.ex. Socialstyrelsens Nationella riktlinjer)**

Fritext

**Inför kommande avsnitt vill vi veta om du känner till följande begrepp:**

**23) Mikroarray**

- Ja
- Nej

**24) Genpanel**

- Ja
- Nej

**25) Helexomsekvensering**

- Ja
- Nej

**26) Helgenomsekvensering**

- Ja
- Nej

**MIKROARRAY**

Frågor i det här avsnittet handlar om diagnostiska tester för att identifiera variationer i kopienummer associerade med sjukdom, så kallade mikroarraytester.

**Hur säker känner du dig kring följande aspekter av mikroarraytester?***Om du inte har utfört en viss uppgift i din yrkesroll, kryssa alternativet "ej relevant":*

**27) Vara medveten om för vilka indikationer man väljer mikroarraytest**

- Mycket osäker
- Ganska osäker
- Ganska säker
- Mycket säker
- Ej relevant

**28) Förklara innebörden av ett mikroarraytest med patienter och/eller familjer inför ett ev. test, t.ex. tekniska aspekter, begränsningar, varianter av oklar betydelse etc.**

- Mycket osäker
- Ganska osäker
- Ganska säker
- Mycket säker
- Ej relevant

**29) Underlätta för patienten att göra informerade val kring mikroarray, t.ex. risker och fördelar, sekundära fynd, påverkan på familjer etc.**

- Mycket osäker
- Ganska osäker
- Ganska säker
- Mycket säker
- Ej relevant

**30) Förstå resultatrapporter från mikroarraytest**

- Mycket osäker
- Ganska osäker
- Ganska säker
- Mycket säker
- Ej relevant

**31) Verifiera rapporter genom att kontrollera litteratur och databaser, t.ex. OMIM, DECIPHER, gnomAD / ExAC etc.**

- Mycket osäker
- Ganska osäker
- Ganska säker
- Mycket säker
- Ej relevant

**32) Förklara och diskutera resultat från mikroarraytest med patienter/familjer**

- Mycket osäker
- Ganska osäker
- Ganska säker
- Mycket säker
- Ej relevant

**33) Har du beställt mikroarraytest under de senaste 12 månaderna som en del av ditt kliniska arbete eller i din roll inom forskning? Välj ett alternativ**

- Ja, på eget initiativ: gå till fråga 34.
- Ja, i samråd med en genetiker: gå till fråga 34.
- Nej: gå till fråga 35.

**34) Hur ofta har du beställt mikroarraytest under de senaste 12 månaderna?**

- Dagligen
- Varje vecka
- Varje månad
- Varje kvartal
- En eller två gånger om året
- Vet ej

**35) Av vilken anledning har du inte beställt mikroarraytest de senaste 12 månaderna?** *Markera alla som gäller*

- Ej behov av genetisk utredning
- Jag remitterade de patienter som kunde behöva mikroarraytest till en klinisk genetisk verksamhet eller annan specialistverksamhet
- Jag kan inte beställa ett mikroarraytest i min nuvarande roll / vid min nuvarande verksamhet/avdelning, t.ex. brist på tillgång till, eller finansiering för, testning
- Jag är inte säker på hur man beställer ett mikroarraytest
- Jag är inte säker på hur relevant mikroarray är för min verksamhet/mina patienter
- Microarraytester är inte relevanta för min verksamhet/mina patienter
- Om annat, specificera

**36) Om du inte kan beställa ett mikroarraytest, förklara varför**

## GENPANELER

Frågor i det här avsnittet handlar om diagnostiska tester för att identifiera varianter i flera väl karakteriserade gener associerade med en viss fenotyp och klinisk presentation, så kallad genpaneltest.

**Hur säker känner du dig kring följande aspekter av genpaneltester?**

*Om du inte har utfört en viss uppgift i din yrkesroll, kryssa alternativet "ej relevant":*

**37) Vara medveten om för vilka indikationer man väljer genpaneltester**

- Mycket osäker
- Ganska osäker
- Ganska säker
- Mycket säker
- Ej relevant

**38) Förklara innebörden av en genpanel med patienter och/eller familjer inför ett ev. test, t.ex. tekniska aspekter, begränsningar, varianter av oklar betydelse etc.**

- Mycket osäker
- Ganska osäker
- Ganska säker
- Mycket säker
- Ej relevant

**39) Att underlätta för patienten att göra informerade val kring genpaneltester, t.ex. risker och fördelar, sekundära fynd, påverkan på familjer etc.**

- Mycket osäker
- Ganska osäker
- Ganska säker
- Mycket säker
- Ej relevant

**40) Förstå resultatrapporter från genpaneltester**

- Mycket osäker
- Ganska osäker
- Ganska säker
- Mycket säker
- Ej relevant

**41) Verifiera rapporter genom att kontrollera litteratur och databaser, t.ex. OMIM, DECIPHER, gnomAD / ExAC etc.**

- Mycket osäker
- Ganska osäker
- Ganska säker
- Mycket säker
- Ej relevant

**42) Förklara och diskutera resultat från genpaneltester med patienter/familjer**

- Mycket osäker
- Ganska osäker
- Ganska säker
- Mycket säker
- Ej relevant

**43) Har du beställt genpaneltest under de senaste 12 månaderna som en del av ditt kliniska arbete eller i din roll inom forskning?**

- Ja, på eget initiativ*: gå till fråga* *44.*
- Ja, i samråd med en genetiker*: gå till fråga* *44.*
- Nej*: gå till fråga* *46.*

**44) Hur ofta har du beställt genpaneltest under de senaste 12 månaderna?**

- Dagligen
- Varje vecka
- Varje månad
- Varje kvartal
- En eller två gånger om året
- Vet ej

**45) Vilken typ av genpaneltest beställde du under de senaste 12 månaderna?**

*Markera alla som gäller*

- Medfödda
- Förvärvade (somatiska)
- Vet ej

**46) Av vilken anledning har du inte beställt genpaneltest de senaste 12 månaderna?** *Markera alla som gäller*

- Ej behov av genetisk utredning
- Jag remitterade de patienter som kunde behöva genpaneltest till en klinisk genetisk verksamhet eller annan specialistverksamhet
- Jag kan inte beställa ett genpaneltest i min nuvarande roll / vid min nuvarande verksamhet/avdelning, t.ex. brist på tillgång till, eller finansiering för, testning
- Jag är inte säker på hur man beställer ett genpaneltest
- Jag är inte säker på hur relevant genpaneltester är för min verksamhet/mina patienter
- Genpaneltester är inte relevanta för min verksamhet/mina patienter
- Om annat, specificera

**47) Om du inte kan beställa ett genpaneltest, förklara varför..**

**HELEXOMSEKVENSERING**

Frågor i det här avsnittet handlar om diagnostiska tester för att bestämma sekvensen för alla exoner (kodande regioner) i ett genom, så kallad helexomsekvensering.

**Hur säker känner du dig kring följande aspekter av helexomsekvensering?**

Om du inte har utfört en viss uppgift i din yrkesroll, kryssa alternativet "ej relevant":

**48) Förstå på vilka indikationer man väljer helexomsekvensering**

- Mycket osäker
- Ganska osäker
- Ganska säker
- Mycket säker
- Ej relevant

**49) Förklara innebörden av helexomsekvensering för patienter och/eller familjer inför ett ev. test, t.ex. tekniska aspekter, begränsningar, varianter av osäker/okänd betydelse etc.**

- Mycket osäker
- Ganska osäker
- Ganska säker
- Mycket säker
- Ej relevant

**50) Att underlätta för patienten att göra informerade val kring helexomsekvensering, t.ex. risker och fördelar, sekundära fynd, påverkan på familjer etc.**

- Mycket osäker
- Ganska osäker
- Ganska säker
- Mycket säker
- Ej relevant

**51) Förstå resultatrapporter från helexomsekvensering**

- Mycket osäker
- Ganska osäker
- Ganska säker
- Mycket säker
- Ej relevant

**52) Verifiera rapporter genom att kontrollera litteratur och databaser, t.ex. OMIM, DECIPHER, gnomAD / ExAC etc.**

- Mycket osäker
- Ganska osäker
- Ganska säker
- Mycket säker
- Ej relevant

**53) Förklara och diskutera resultat från helexomsekvensering med patienter/familjer**

- Mycket osäker
- Ganska osäker
- Ganska säker
- Mycket säker
- Ej relevant

**54) Har du beställt tester med helexomsekvensering under de senaste 12 månaderna som en del av ditt kliniska arbete eller i din roll inom forskning?**

- Ja, på eget initiativ*: gå till fråga* *55.*
- Ja, i samråd med en genetiker*: gå till fråga* *55.*
- Nej*: gå till fråga* *57.*

**55) Hur ofta har du beställt helexomsekvensering under de senaste 12 månaderna?**

- Dagligen
- Varje vecka
- Varje månad
- Varje kvartal
- En eller två gånger om året
- Vet ej

**56) Vilken typ av helexomsekvensering beställde du under de senaste 12 månaderna?**

*Markera alla som gäller*

- Medfödda
- Förvärvade (somatiska)
- Vet ej

**57) Av vilken anledning har du inte beställt helexomsekvensering de senaste 12 månaderna?** *Markera alla som gäller*

- Ej behov av genetisk utredning
- Jag remitterade de patienter som kunde behöva helexomsekvensering till en klinisk genetisk verksamhet eller annan specialistverksamhet
- Jag kan inte beställa ett test med helexomsekvensering i min nuvarande roll / vid min nuvarande verksamhet/avdelning, t.ex. brist på tillgång till, eller finansiering för, testning
- Jag är inte säker på hur man beställer helexomsekvensering
- Jag är inte säker på hur relevant helexomsekvensering är för min verksamhet/mina patienter
- Helexomsekvensering är inte relevanta för min verksamhet/mina patienter
- Om annat, specificera

**58) Om du inte kan beställa ett helexomsekvensering, förklara varför..**

**HELGENOMSEKVENSERING**

Frågor i det här avsnittet handlar om diagnostiska tester för att bestämma sekvensen för hela genomet (kodande och icke-kodande regioner), så kallad helgenomsekvensering.

**Hur säker känner du dig kring följande aspekter av helgenomsekvensering?**

*Om du inte har utfört en viss uppgift i din yrkesroll, kryssa alternativet "ej relevant":*

**59) Förstå på vilka indikationer man väljer helgenomsekvensering**

- Mycket osäker
- Ganska osäker
- Ganska säker
- Mycket säker
- Ej relevant

**60) Förklara innebörden av helgenomsekvensering för patienter och/eller familjer inför ett ev. test, t.ex. tekniska aspekter, begränsningar, varianter av osäker/okänd betydelse etc.**

- Mycket osäker
- Ganska osäker
- Ganska säker
- Mycket säker
- Ej relevant

**61) Att underlätta för patienten att göra informerade val kring helgenomsekvensering, t.ex. risker och fördelar, sekundära fynd, påverkan på familjer etc.**

- Mycket osäker
- Ganska osäker
- Ganska säker
- Mycket säker
- Ej relevant

**62) Förstå resultatrapporter från helgenomsekvensering**

- Mycket osäker
- Ganska osäker
- Ganska säker
- Mycket säker
- Ej relevant

**63) Verifiera rapporter genom att kontrollera litteratur och databaser, t.ex. OMIM, DECIPHER, gnomAD / ExAC etc.**

- Mycket osäker
- Ganska osäker
- Ganska säker
- Mycket säker
- Ej relevant

**64) Förklara och diskutera resultat från helgenomsekvensering med patienter/familjer**

- Mycket osäker
- Ganska osäker
- Ganska säker
- Mycket säker
- Ej relevant

**65) Har du beställt tester med helgenomsekvensering under de senaste 12 månaderna som en del av ditt kliniska arbete eller i din roll inom forskning?**

- Ja, på eget initiativ*: gå till fråga* *66.*
- Ja, i samråd med en genetiker*: gå till fråga* *66.*
- Nej*: gå till fråga* *68.*

**66) Hur ofta har du beställt helgenomsekvensering under de senaste 12 månaderna?**

- Dagligen
- Varje vecka
- Varje månad
- Varje kvartal
- En eller två gånger om året
- Vet ej

**67) Vilken typ av helgenomsekvensering beställde du under de senaste 12 månaderna?**

*Markera alla som gäller*

- Medfödda
- Förvärvade (somatiska)
- Vet ej

**68) Av vilken anledning har du inte beställt helgenomsekvensering de senaste 12 månaderna?** *Markera alla som gäller*

- Ej behov av genetisk utredning
- Jag remitterade de patienter som kunde behöva helgenomsekvensering till en klinisk genetisk verksamhet eller annan specialistverksamhet
- Jag kan inte beställa ett test med helgenomsekvensering i min nuvarande roll / vid min nuvarande verksamhet/avdelning, t.ex. brist på tillgång till, eller finansiering för, testning
- Jag är inte säker på hur man beställer helgenomsekvensering
- Jag är inte säker på hur relevant helgenomsekvensering är för min verksamhet/mina patienter
- Helgenomsekvensering är inte relevanta för min verksamhet/mina patienter
- Om annat, specificera

**69) Om du inte kan beställa ett helgenomsekvensering, förklara varför..**

**Nedan finns en lista över några av de olika steg som ingår i samband med ett genomiskt test (helexom- ellerhelgenomsekvensering), från steg inför testet (pre-test) till steg efter testet (post-test). Ange vilka steg/uppgifter du för närvarandeutför och vilka du förväntar dig att du kommer att utföra i framtiden om du har tillräcklig kompetens, utbildning och stöd.** Välj ett alternativ per rad.

Obs: denna fråga avser INTE mikroarray- eller genpaneltester. Vi frågar bara om helexom- och helgenomsekvensering i denna fråga.

Vi är medvetna om att inte all exom/genomsekvensering följer samma process. Dessa steg är bara riktlinjer; använd rutan "Kommentar" nedan för att beskriva andra steg du utför eller förväntar dig att utföra.

**Pre-test (innan ett genomiskt test):**

**70) Inhämtar information om genetiska tillstånd som en del av en familje- eller medicinsk historia**

- Utför nu och kommer att fortsätta utföra
- Utför nu men förväntar inte att utföra längre fram
- Utför inte nu men förväntas göra det längre fram
- Utför inte nu och förväntar inte att göra det längre fram
- Osäker
- Ej relevant

**71) Bedömer om en patient är relevant för ett genomiskt test**

- Utför nu och kommer att fortsätta utföra
- Utför nu men förväntar inte att utföra längre fram
- Utför inte nu men förväntas göra det längre fram
- Utför inte nu och förväntar inte att göra det längre fram
- Osäker
- Ej relevant

**72) Ger genetisk vägledning innan ett test (pre-test) för att hjälpa patienten att fatta ett välgrundat beslut, t.ex. om genetik, begränsningar för tester, varianter av osäker/okänd betydelse, sekundära fynd, eller konsanguinitet (släktgifte)**

- Utför nu och kommer att fortsätta utföra
- Utför nu men förväntar inte att utföra längre fram
- Utför inte nu men förväntas göra det längre fram
- Utför inte nu och förväntar inte att göra det längre fram
- Osäker
- Ej relevant

**73) Beställer ett genomiskt test för en patient**

- Utför nu och kommer att fortsätta utföra
- Utför nu men förväntar inte att utföra längre fram
- Utför inte nu men förväntas göra det längre fram
- Utför inte nu och förväntar inte att göra det längre fram
- Osäker
- Ej relevant

**Test och testning:**

**74) Deltar i multidisciplinärt team-möten för att diskutera genomiskt test**

- Utför nu och kommer att fortsätta utföra
- Utför nu men förväntar inte att utföra längre fram
- Utför inte nu men förväntas göra det längre fram
- Utför inte nu och förväntar inte att göra det längre fram
- Osäker
- Ej relevant

**75) Bistår laboratoriet att begränsa/välja ut gener av intresse (skapa en genlista för att prioritera variantanalys)**

- Utför nu och kommer att fortsätta utföra
- Utför nu men förväntar inte att utföra längre fram
- Utför inte nu men förväntas göra det längre fram
- Utför inte nu och förväntar inte att göra det längre fram
- Osäker
- Ej relevant

**76) Tillhandahåller fenotypisk information till laboratoriet för att de ska kunna prioritera**

**variantanalys**

- Utför nu och kommer att fortsätta utföra
- Utför nu men förväntar inte att utföra längre fram
- Utför inte nu men förväntas göra det längre fram
- Utför inte nu och förväntar inte att göra det längre fram
- Osäker
- Ej relevant

**77) Söker i litteraturen och databaser för bevis för patogenicitet av variant**

- Utför nu och kommer att fortsätta utföra
- Utför nu men förväntar inte att utföra längre fram
- Utför inte nu men förväntas göra det längre fram
- Utför inte nu och förväntar inte att göra det längre fram
- Osäker
- Ej relevant

**78) Deltar i multidisciplinära team-möten för att diskutera variantprioritering , tolkning och klassificering**

- Utför nu och kommer att fortsätta utföra
- Utför nu men förväntar inte att utföra längre fram
- Utför inte nu men förväntas göra det längre fram
- Utför inte nu och förväntar inte att göra det längre fram
- Osäker
- Ej relevant

**Post-test (efter genomtestning):**

**79) Delger testresultat till patienter / familjer**

- Utför nu och kommer att fortsätta utföra
- Utför nu men förväntar inte att utföra längre fram
- Utför inte nu men förväntas göra det längre fram
- Utför inte nu och förväntar inte att göra det längre fram
- Osäker
- Ej relevant

**80) *Ger genetisk vägledning till patienter / familjer, t.ex. förklara varianter av oklar betydelse, sekundära fynd, eller konsanginuitet (släktgifte)***

- Utför nu och kommer att fortsätta utföra
- Utför nu men förväntar inte att utföra längre fram
- Utför inte nu men förväntas göra det längre fram
- Utför inte nu och förväntar inte att göra det längre fram
- Osäker
- Ej relevant

**81) Organiserar för, eller remittera till, ytterligare tester av familjemedlemmar om det behövs, t.ex. kaskadtestning eller segregationsanalyser**

- Utför nu och kommer att fortsätta utföra
- Utför nu men förväntar inte att utföra längre fram
- Utför inte nu men förväntas göra det längre fram
- Utför inte nu och förväntar inte att göra det längre fram
- Osäker
- Ej relevant

**82) Ansvarar för fortsatt handläggning av patienten, t.ex. informerar om återupprepningsrisker och diskuterar möjliga alternativ för och planering av reproduktion**

- Utför nu och kommer att fortsätta utföra
- Utför nu men förväntar inte att utföra längre fram
- Utför inte nu men förväntas göra det längre fram
- Utför inte nu och förväntar inte att göra det längre fram
- Osäker
- Ej relevant

**83) Följer upp patienten efter testet för att kontrollera förståelsen av resultatet samt att ställa ytterligare frågor**

- Utför nu och kommer att fortsätta utföra
- Utför nu men förväntar inte att utföra längre fram
- Utför inte nu men förväntas göra det längre fram
- Utför inte nu och förväntar inte att göra det längre fram
- Osäker
- Ej relevant

**84) Förklara av vilken anledning du inte förväntar dig att utföra de valda stegen:**

**85) Om du fick välja, hur skulle du föredra att genomiska tester och resultat handläggs**

**i din klinik/ yrkesutövning? *Välj ett svar nedan***

- Du initierar testning och diskuterar resultat med patienter / familjer
- Du initierar testning och diskuterar resultat med patienter / familjer, med stöd från en klinisk genetisk avdelning efter behov
- Du hänvisar till en klinisk genetisk avdelning för att inleda testning och diskutera resultat med patienter / familjer
- Du har tillgång till medarbetare med utökad kunskap om genetik/genomik och som har koppling till klinisk genetik, t.ex. genetisk vägledare
- Du träffar inte och förväntar dig inte att träffa patienter som skulle ha nytta av genomtestning
- Vet ej

**86) Om du behöver stöd, ange inom vilka områden som det kan vara mest värdefullt.**

***Välj alla som passer***

- Råd om huruvida ett genomiskt test är lämpligt 85
- Genetisk vägledning inför ev. testning
- Samtycke
- Tolka resultat
- Medarbetare med extra kompetens kring genomik (t.ex genetisk vägledare) med direkt koppling till klinisk genetik
- Diskutera resultat med familjer
- Uppföljande genetisk vägledning av familjen

**87) Vänligen ge fler kommentarer om du vill klargöra, t.ex. detaljer om stöd, diskussion mellan olika discipliner för att få stöd.**

**88) Vilka faktorer påverkar din föredragna modell för att delge genomiska testresultat?**

**Nedan är en lista över olika sätt som genomsekvensering och andra genomiska**

**tester kan initieras och diskuteras med patienter. Ange vilka som du för**

**närvarande genomför i din verksamhet och/eller som du tror att du kommer att göra**

**oftare de närmaste fem åren. *Markera alla som gäller i varje kolumn.***

**89) Läkaren efterfrågar genomiska tester för att underlätta för diagnos/prognos/ behandling/pågående handläggning av patienten**

- Förekommer nu och kommer ej att förändras
- Förekommer nu och kommer att ske mer frekvent de närmaste 5 åren
- Förekommer inte nu men kommer att ske mer frekvent de närmaste 5 åren
- Förekommer inte nu och kommer ej att förändras
- Vet ej

**90) Läkaren efterfrågar farmakogenomiska test (genetisk test som analyserar specifikt utvalda gener som kan påverka effekten av vissa för patienten aktuella läkemedel) för att underlätta behandlingen**

- Förekommer nu och kommer ej att förändras
- Förekommer nu och kommer att ske mer frekvent de närmaste 5 åren
- Förekommer inte nu men kommer att ske mer frekvent de närmaste 5 åren
- Förekommer inte nu och kommer ej att förändras
- Vet ej

**91) Läkaren remitterar för genomiska tester för att underlätta för diagnos/prognos/behandling/pågående handläggning av patienten, inklusive farmakogenomiska tester**

- Förekommer nu och kommer ej att förändras
- Förekommer nu och kommer att ske mer frekvent de närmaste 5 åren
- Förekommer inte nu men kommer att ske mer frekvent de närmaste 5 åren
- Förekommer inte nu och kommer ej att förändras
- Vet ej

**92) Patienter/familjer frågar om genomiska tester för att underlätta diagnos/prognos/behandling/pågående handläggning**

- Förekommer nu och kommer ej att förändras
- Förekommer nu och kommer att ske mer frekvent de närmaste 5 åren
- Förekommer inte nu men kommer att ske mer frekvent de närmaste 5 åren
- Förekommer inte nu och kommer ej att förändras
- Vet ej

**93) Patienter/familjer frågar om direct-to-consumer tester/ personliga genomiska test och/eller DNA-testning online , som t.ex. SmartDNA eller 23 & Me**

- Förekommer nu och kommer ej att förändras
- Förekommer nu och kommer att ske mer frekvent de närmaste 5 åren
- Förekommer inte nu men kommer att ske mer frekvent de närmaste 5 åren
- Förekommer inte nu och kommer ej att förändras
- Vet ej

**94) Tror du att ökad kunskap om genomik och användning av genomiska tester**

**kommer att påverka din verksamhet de närmaste åren?**

- 0-2 år
- 3-5 år
- Mer än 5 år
- Vet ej

**95) Varför?**

**96) Vilka områden kommer att påverkas? *Markera alla som gäller.***

- Hur jag arbetar i min roll som läkare
- Min arbetsbelastning
- Handläggningen av patienten
- Om annat, specificera

**97) Vänligen ge fler kommentarer om du vill förtydliga.**

**98) Känner du dig förberedd för att använda tester med genomsekvensering i din verksamhet?**

- Ja
- Nej
- Vet ej

**99) Förtydliga ditt svar. T.ex. vad kan till exempel behöva förändras för att hjälpa dig att känna dig förberedd?**

## Avsnitt 3: Utbildning – Nuvarande och framtida

Följande frågor gäller tidigare och framtida professionell utveckling.

**I din yrkesroll, hur trygg är du i din….**

**100) Kunskap om genetik och genomik**

- Mycket osäker
- Ganska osäker
- Ganska säker
- Mycket säker
- Vet ej

**101) Förmåga att ta fram information om patientens genetiska tillstånd som en del av en patient- och familjeanamnes**

- Mycket osäker
- Ganska osäker
- Ganska säker
- Mycket säker
- Vet ej

**102) Förmåga att förklara genetiska och genomiska koncept för patienter (t.ex. ärftlighet, testning, riskbedömning)**

- Mycket osäker
- Ganska osäker
- Ganska säker
- Mycket säker
- Vet ej

**103) Förmåga att fatta beslut kring vidare handläggning baserat på genomisk information**

- Mycket osäker
- Ganska osäker
- Ganska säker
- Mycket säker
- Vet ej

**104) Vad skulle kunna hjälpa dig att förbättra din förmåga ytterligare?**

**105) Skulle en utökad kunskap inom genomisk medicin förändra hur du utför ditt jobb?**

Ja

Nej

Vet ej

**106) Vänligen förklara hur…**

**107) Vänligen förklara varför…**

**108) Har du deltagit i någon kompetensutveckling inom genomik det senaste året, som**

**till exempel föreläsningar, seminarier, workshops eller kurser, antingen på plats**

**eller online?**

- Ja
- Nej

**109) Vänligen ange vilken typ av kompetensutveckling...**

**110) Har du tillhandahållit/givit någon kompetensutveckling inom genomik det senaste året, som till exempel föreläsningar, seminarier, workshops eller kurser, antingen på plats eller online?**

- Ja
- Nej

**111) Vänligen ange vilken typ av kompetensutveckling…**

**112) Tycker du att du har fått tillräcklig utbildning inom genetik/genomik i din**

**grundutbildning?**

- Ja
- Nej

**113) När anser du att utökad utbildning inom genomik ska erhållas?**

- Under grundutbildningen
- Som en del av kompetensutveckling under yrkesutövning

**Nedan är en lista över aktiviteter för att hålla sig uppdaterad med, eller lära sig nya färdigheter, inom genomisk medicin. Ange vilka aktiviteter du för närvarande deltar i och/eller föredrar att delta i.** *Välj ett alternativ per rad.*

**114) Praktik eller auskultera i ett laboratorium som utför genomiska analyser eller vid en klinisk genetisk mottagning**

- Deltar för närvarande i och kommer fortsätta delta
- Deltar för närvarande i men skulle föredra att inte delta
- Deltar inte för närvarande i men skulle föredra att delta
- Deltar inte för närvarande i och skulle inte delta
- Osäker
- Ej relevant

**115) Forskningsprojekt inom genomik, t.ex. samarbete med forskningslaboratorium**

- Deltar för närvarande i och kommer fortsätta delta
- Deltar för närvarande i men skulle föredra att inte delta
- Deltar inte för närvarande i men skulle föredra att delta
- Deltar inte för närvarande i och skulle inte delta
- Osäker
- Ej relevant

**116) Multidisciplinära möten**

- Deltar för närvarande i och kommer fortsätta delta
- Deltar för närvarande i men skulle föredra att inte delta
- Deltar inte för närvarande i men skulle föredra att delta
- Deltar inte för närvarande i och skulle inte delta
- Osäker
- Ej relevant

**117) Interna seminarier, konferenser, etc inom genetik eller genomik för arbetsplatsen**

**Interna**

- Deltar för närvarande i och kommer fortsätta delta
- Deltar för närvarande i men skulle föredra att inte delta
- Deltar inte för närvarande i men skulle föredra att delta
- Deltar inte för närvarande i och skulle inte delta
- Osäker
- Ej relevant

**118) Externa seminarier, konferenser, etc inom genetik eller genomik**

- Deltar för närvarande i och kommer fortsätta delta
- Deltar för närvarande i men skulle föredra att inte delta
- Deltar inte för närvarande i men skulle föredra att delta
- Deltar inte för närvarande i och skulle inte delta
- Osäker
- Ej relevant

**119) Externa kurser inom genetik eller genomik**

- Deltar för närvarande i och kommer fortsätta delta
- Deltar för närvarande i men skulle föredra att inte delta
- Deltar inte för närvarande i men skulle föredra att delta
- Deltar inte för närvarande i och skulle inte delta
- Osäker
- Ej relevant

**120) Universitetskurser (hp givande)**

- Deltar för närvarande i och kommer fortsätta delta
- Deltar för närvarande i men skulle föredra att inte delta
- Deltar inte för närvarande i men skulle föredra att delta
- Deltar inte för närvarande i och skulle inte delta
- Osäker
- Ej relevant

**121) Online webbinarier, kurser, MOOCs (Massive, open, online course), etc.**

- Deltar för närvarande i och kommer fortsätta delta
- Deltar för närvarande i men skulle föredra att inte delta
- Deltar inte för närvarande i men skulle föredra att delta
- Deltar inte för närvarande i och skulle inte delta
- Osäker
- Ej relevant

**122) Läsa texter inom specialområde (tidskrifter, artiklar, böcker, etc.)**

- Deltar för närvarande i och kommer fortsätta delta
- Deltar för närvarande i men skulle föredra att inte delta
- Deltar inte för närvarande i men skulle föredra att delta
- Deltar inte för närvarande i och skulle inte delta
- Osäker
- Ej relevant

**123) Avsatt tid för studier på arbetstid**

- Deltar för närvarande i och kommer fortsätta delta
- Deltar för närvarande i men skulle föredra att inte delta
- Deltar inte för närvarande i men skulle föredra att delta
- Deltar inte för närvarande i och skulle inte delta
- Osäker
- Ej relevant

**124) Handledarledd undervisning i mindre grupper**

- Deltar för närvarande i och kommer fortsätta delta
- Deltar för närvarande i men skulle föredra att inte delta
- Deltar inte för närvarande i men skulle föredra att delta
- Deltar inte för närvarande i och skulle inte delta
- Osäker
- Ej relevant

**125) Presentationer på avdelningen**

- Deltar för närvarande i och kommer fortsätta delta
- Deltar för närvarande i men skulle föredra att inte delta
- Deltar inte för närvarande i men skulle föredra att delta
- Deltar inte för närvarande i och skulle inte delta
- Osäker
- Ej relevant

**126) Konsultera kollegor**

- Deltar för närvarande i och kommer fortsätta delta
- Deltar för närvarande i men skulle föredra att inte delta
- Deltar inte för närvarande i men skulle föredra att delta
- Deltar inte för närvarande i och skulle inte delta
- Osäker
- Ej relevant

**127) Massmedia (TV, tidningar)**

- Deltar för närvarande i och kommer fortsätta delta
- Deltar för närvarande i men skulle föredra att inte delta
- Deltar inte för närvarande i men skulle föredra att delta
- Deltar inte för närvarande i och skulle inte delta
- Osäker
- Ej relevant

**128) Sociala medier, t.ex., twitter journal club, Poddar**

- Deltar för närvarande i och kommer fortsätta delta
- Deltar för närvarande i men skulle föredra att inte delta
- Deltar inte för närvarande i men skulle föredra att delta
- Deltar inte för närvarande i och skulle inte delta
- Osäker
- Ej relevant

**Vilka områden inom genetik/genomik har du idag kunskap om och vilka skulle du vilja lära dig mer om i framtiden? *Välj ett alternativ per rad.***

**129) Grundläggande koncept**

- Har kunskap om och önskar lära mer
- Har kunskap om och ser inget behov av att lära mer
- Har inte kunskap om men önskar lära mer
- Har inte kunskap om och ser inget behov av att lära mer
- Osäker
- Ej relevant

**130) Avvikelser och sjukdomar**

- Har kunskap om och önskar lära mer
- Har kunskap om och ser inget behov av att lära mer
- Har inte kunskap om men önskar lära mer
- Har inte kunskap om och ser inget behov av att lära mer
- Osäker
- Ej relevant

**131) Tillämpningar av genomisk medicin som används idag**

- Har kunskap om och önskar lära mer
- Har kunskap om och ser inget behov av att lära mer
- Har inte kunskap om men önskar lära mer
- Har inte kunskap om och ser inget behov av att lära mer
- Osäker
- Ej relevant

**132) Framväxande tillämpningar inom genomisk medicin**

- Har kunskap om och önskar lära mer
- Har kunskap om och ser inget behov av att lära mer
- Har inte kunskap om men önskar lära mer
- Har inte kunskap om och ser inget behov av att lära mer
- Osäker
- Ej relevant

**Vilka områden inom genetisk/genomisk testning och teknologier har du idag**

**kunskap om och vilka skulle du vilja lära dig mer om i framtiden? *Välj ett alternativ per rad.***

**133) Olika typer av genetiska tester, t.ex. mikroarray, enstaka gentest**

- Har kunskap om och önskar lära mer
- Har kunskap om och ser inget behov av att lära mer
- Har inte kunskap om men önskar lära mer
- Har inte kunskap om och ser inget behov av att lära mer
- Osäker
- Ej relevant

**134) Olika typer av genomiska tester, t.ex. paneler, helexom-/helgenom-sekvensering**

- Har kunskap om och önskar lära mer
- Har kunskap om och ser inget behov av att lära mer
- Har inte kunskap om men önskar lära mer
- Har inte kunskap om och ser inget behov av att lära mer
- Osäker
- Ej relevant

**135) Olika tillämpningar av genomiska tester vid förvärvade/somatiska förändringar**

- Har kunskap om och önskar lära mer
- Har kunskap om och ser inget behov av att lära mer
- Har inte kunskap om men önskar lära mer
- Har inte kunskap om och ser inget behov av att lära mer
- Osäker
- Ej relevant

**136) Olika tillämpningar av genomiska tester vid konstitutionella/medfödda förändringar**

- Har kunskap om och önskar lära mer
- Har kunskap om och ser inget behov av att lära mer
- Har inte kunskap om men önskar lära mer
- Har inte kunskap om och ser inget behov av att lära mer
- Osäker
- Ej relevant

**137) Klinisk nytta av tester, t.ex. diagnos/ prognos/behandling/ pågående hantering, inclusive farmakogenomik (en genetisk test som analyserar specifikt utvalda gener som kan påverka effekten av vissa för patienten aktuella läkemedel.)**

- Har kunskap om och önskar lära mer
- Har kunskap om och ser inget behov av att lära mer
- Har inte kunskap om men önskar lära mer
- Har inte kunskap om och ser inget behov av att lära mer
- Osäker
- Ej relevant

**138) Klassificering av genomiska varianter som kan påvisas vid testning**

- Har kunskap om och önskar lära mer
- Har kunskap om och ser inget behov av att lära mer
- Har inte kunskap om men önskar lära mer
- Har inte kunskap om och ser inget behov av att lära mer
- Osäker
- Ej relevant

**139) Begränsningar av testning, t.ex. vilka typer av avvikelser som inte kan upptäckas av testet**

- Har kunskap om och önskar lära mer
- Har kunskap om och ser inget behov av att lära mer
- Har inte kunskap om men önskar lära mer
- Har inte kunskap om och ser inget behov av att lära mer
- Osäker
- Ej relevant

**Vilka områden inom handläggning före och efter genomisk testning har du idag kunskap om och vilka skulle du vilja lära dig mer om i framtiden? *Välj ett alternativ per rad.***

**140) Kan identifiera patienter som kan dra nytta av genomisk testning**

- Har kunskap om och önskar lära mer
- Har kunskap om och ser inget behov av att lära mer
- Har inte kunskap om men önskar lära mer
- Har inte kunskap om och ser inget behov av att lära mer
- Osäker
- Ej relevant

**141) Kommunicera med patienter om genomiska tester**

- Har kunskap om och önskar lära mer
- Har kunskap om och ser inget behov av att lära mer
- Har inte kunskap om men önskar lära mer
- Har inte kunskap om och ser inget behov av att lära mer
- Osäker
- Ej relevant

**142) Göra genetiska riskbedömningar (t.ex. baserat på familjehistoria, nedärvningsmönster och/eller resultat från genetiska tester)**

- Har kunskap om och önskar lära mer
- Har kunskap om och ser inget behov av att lära mer
- Har inte kunskap om men önskar lära mer
- Har inte kunskap om och ser inget behov av att lära mer
- Osäker
- Ej relevant

**143) Remittera för ett genomiskt test på korrekt sätt**

- Har kunskap om och önskar lära mer
- Har kunskap om och ser inget behov av att lära mer
- Har inte kunskap om men önskar lära mer
- Har inte kunskap om och ser inget behov av att lära mer
- Osäker
- Ej relevant

**144) Beställa ett genomiskt test för en patient, t.ex. patologiformulär, rör för blod, samtycke etc. på korrekt sätt**

- Har kunskap om och önskar lära mer
- Har kunskap om och ser inget behov av att lära mer
- Har inte kunskap om men önskar lära mer
- Har inte kunskap om och ser inget behov av att lära mer
- Osäker
- Ej relevant

**145) Tolka genomiska testresultat**

- Har kunskap om och önskar lära mer
- Har kunskap om och ser inget behov av att lära mer
- Har inte kunskap om men önskar lära mer
- Har inte kunskap om och ser inget behov av att lära mer
- Osäker
- Ej relevant

**146) Identifiera ytterligare familjemedlemmar som kan dra nytta av genomisk testning**

**(kaskadtestning)**

- Har kunskap om och önskar lära mer
- Har kunskap om och ser inget behov av att lära mer
- Har inte kunskap om men önskar lära mer
- Har inte kunskap om och ser inget behov av att lära mer
- Osäker
- Ej relevant

**Vilka områden inom etiska, juridiska och sociala konsekvenser (ELSI) av genetisk/genomisk testning har du idag kunskap om och vilka skulle du vilja lära dig mer om i framtiden? *Välj ett alternativ per rad.***

**147) Etiska konsekvenser, t.ex. oväntade fynd, varianter av osäker/okänd betydelse etc.**

- Har kunskap om och önskar lära mer
- Har kunskap om och ser inget behov av att lära mer
- Har inte kunskap om men önskar lära mer
- Har inte kunskap om och ser inget behov av att lära mer
- Osäker
- Ej relevant

**148) Juridiska konsekvenser, t.ex. datadelning, integritet, sekretess, försäkring, etc.**

- Har kunskap om och önskar lära mer
- Har kunskap om och ser inget behov av att lära mer
- Har inte kunskap om men önskar lära mer
- Har inte kunskap om och ser inget behov av att lära mer
- Osäker
- Ej relevant

**149) Psykosociala konsekvenser, t.ex. kommunicera information till familj, reproduktionsalternativ, förmedla patienter till stödgrupper, etc.**

- Har kunskap om och önskar lära mer
- Har kunskap om och ser inget behov av att lära mer
- Har inte kunskap om men önskar lära mer
- Har inte kunskap om och ser inget behov av att lära mer
- Osäker
- Ej relevant

**150) Ange andra ämnen inom genomik som du vill få utbildning om…**

**151) Vilken kunskapsnivå kring genetik och genomik upplever du att dina patienter har?**

- Ingen kunskap
- Låg kunskapsnivå
- Neutral
- Hög kunskapsnivå
- Mycket hög kunskapsnivå
- Vet ej

**Nedan följer några påstående om olika antaganden som personer kan ha kring genetisk diagnostik. Hur tror du att din genomsnittliga patient skulle svara på dessa påståenden?**

**152) En genetisk diagnos kan alltid erhållas med genomisk testning.**

- Instämmer inte alls
- Instämmer delvis
- Instämmer helt
- Vet ej

**153) En genetisk diagnos skulle kunna försämra bemötandet i vården.**

- Instämmer inte alls
- Instämmer delvis
- Instämmer helt
- Vet ej

**154) En genetisk diagnos skulle kunna försämra stödet i samhället.**

- Instämmer inte alls
- Instämmer delvis
- Instämmer helt
- Vet ej

**155) En genetisk diagnos skulle kunna förbättra bemötandet i vården.**

- Instämmer inte alls
- Instämmer delvis
- Instämmer helt
- Vet ej

**156) En genetisk diagnos skulle kunna förbättra stödet i samhället.**

- Instämmer inte alls
- Instämmer delvis
- Instämmer helt
- Vet ej

**157) En genetisk diagnos skulle förbättra möjligheteten för behandling.**

- Instämmer inte alls
- Instämmer delvis
- Instämmer helt
- Vet ej

**158) Genetisk information är annorlunda och känsligare än annan hälsoinformation.**

- Instämmer inte alls
- Instämmer delvis
- Instämmer helt
- Vet ej

**159) En genetisk diagnos är känsligare information än annan hälsodata då det kan påvisa eventuella framtida sjukdomar.**

- Instämmer inte alls
- Instämmer delvis
- Instämmer helt
- Vet ej

**160) En genetisk diagnos kan öka risken för stigmatisering.**

- Instämmer inte alls
- Instämmer delvis
- Instämmer helt
- Vet ej

**161) En genetisk diagnos kan minska risken för stigmatisering.**

- Instämmer inte alls
- Instämmer delvis
- Instämmer helt
- Vet ej

**162) En genetisk diagnos är känsligare information än annan hälsodata pga att den kan beröra hela familjen och släkten.**

- Instämmer inte alls
- Instämmer delvis
- Instämmer helt
- Vet ej

**163) En genetisk diagnos kan öka risken att obehöriga (t.ex. arbetsgivare eller försäkringbolag) får del av genetisk information.**

- Instämmer inte alls
- Instämmer delvis
- Instämmer helt
- Vet ej

**164) Varifrån upplever du att de flesta av dina patienter fått sin kunskap och åsikter**

**kring genetik /genomik?**

- Skola (grundskola/gymnasium)
- Vidare utbildning (Högskola/Universitet)
- I kontakt med sjukvård
- Anhöriga som varit i kontakt med vården kring detta
- Media (Nyheter/faktaprogram)
- Tv-serier (Fiktiva serier såsom deckare/sjukhusserier etc)
- Sociala medier
- Google eller andra sökmotorer
- Vet ej
- Om annat, specificera

**165) Upplever du att det finns anledningar till att personer inte söker vård som kan leda**

**till en genetisk diagnos?**

- Ja
- Nej
- Vet ej

**166) Vilken/vilka? Du kan välja flera alternativ.**

- Generell okunskap om genetik och genetiska faktorer
- Attityden att genetisk/genomisk information är mer privat än annan hälsoinformation
- Oro för stigmatisering på grund av genetisk diagnos (exempelvis arbetsgivare, försäkringar etc.)
- Att man inte vet var i vården man kan vända sig
- Att patienten inte får hjälp att bli vidareremitterad från till exempel primärvård eller annan specialitet
- Om annat, specificera

**167) Vad tror du skulle kunna underlätta för patienter att komma över dessa hinder?**

**168) Använder du idag något informationsmaterial (skriftliga broschyrer/videos/annat)**

**när du ska prata med dina patienter om genetik/genomik?**

- Ja
- Nej

**169) I så fall vad/vilket?**

**170) Om inte, varför?**

- Behöver inte något underlag när jag informerar
- Känner inte till något material
- Vet inte vilket som är säkert att använda/granskat
- Har inte tillräcklig kunskap för att informera utifrån befintligt material
- Om annat, specificera

**171) Skulle du önska att det fanns ytterligare material att använda som underlag i samtal**

**med dina patienter?**

- Ja
- Nej

**172) I så fall kring:**

- Generellt om genetik/genomik
- Specifikt om genomisk testning
- Nytta och risker relaterat till genomiska tester
- Information kring olika resultat /fynd som genomiska tester kan leda till
- Specifikt om enskilda tillstånd och genetiska faktorer relaterat dessa
- Information om och hjälp för att ta erhålla samtycke inför genomiska tester
- Om annat, specificera

**173) I vilken form?**

- Skriftligt (mer utförliga broschyrer)
- Videoklipp
- Interaktiva beslutsstöd
- Appar
- Chat bots (AI)
- Personlig support (via telefon, mejl eller chat)
- Om annat, specificera

**174) Om du ska hänvisa dina patienter att läsa mer om genetik/genomik på nätet, vilken hemsida hänvisar du då till?**
