# Supplementary material for: Self-assessed knowledge of genomic medicine among non-genetics physicians – results from a nationwide Swedish survey
Source: J Community Genet. 2025 Jul 18;16(6):669–77. doi: 10.1007/s12687-025-00818-y (PMC12569223; doi:10.1007/s12687-025-00818-y)
Supplement: Supplementary file 2 — Supplementary Material 2 [file 12687_2025_818_MOESM2_ESM.docx]

|  | Reports having knowledge of | Reports desire to learn more |
| --- | --- | --- |
| Basic concepts | 75,9% | 77,4% |
| Disorders and diseases | 69,9% | 83,5% |
| Current applications in genomic medicine | 63,9% | 84,2% |
| Emerging applications in genomic medicine | 58,6% | 86,5% |
| Genetic tests, e.g., microarray, single gene tests | 51,1% | 69,2% |
| Genomic tests, e.g., panels, whole exome/genome sequencing | 54,1% | 74,4% |
| Somatic genomic tests | 48,9% | 73,7% |
| Germline genomic tests | 46,6% | 68,4% |
| Clinical utility of tests | 51,9% | 76,7% |
| Classification of genomic data during testing | 47,4% | 70,7% |
| Limitations of testing, e.g., what types of mutations are not detected by tests | 48,9% | 75,9% |
| Recognising patients who may benefit from genomic testing | 60,2% | 70,7% |
| Communication skills with patients | 54,1% | 60,9% |
| Performing genetic risk assessments | 46,6% | 57,9% |
| Referring appropriately for a genomic test | 53,4% | 63,9% |
| Requesting a genomic test for a patient | 49,6% | 55,6% |
| Interpreting genomic test results | 43,6% | 63,2% |
| Identifying additional family members who may benefit from genomic testing (cascade testing) | 34,6% | 53,4% |
| Ethical implications | 43,6% | 67,7% |
| Legal implications | 35,3% | 66,9% |
| Psychosocial implications | 39,8% | 63,2% |

**Table 2* (Full table): All categories of self-assessed knowledge and desire to learn more (n=133)**
